# Supplementary material for: Identification of a novel toxicophore in anti-cancer chemotherapeutics that targets mitochondrial respiratory complex I
Source: eLife. 2020 May 20;9:e55845. doi: 10.7554/eLife.55845 (PMC7316505; doi:10.7554/eLife.55845)
Supplement: Supplementary file 2. — The Ca2+ ion channel binding assay to test the activity of CAI (9), 10, 11, mubritinib (1) and 6 was performed by Eurofins. The percentage inhibition of ion channel was calculated relative to the positive control (1,4,5-IP3). On the scale used a score of 1 = no binding and a score of 100 = binding. The data show that there is no direct binding of these drugs to the ion channels. [file elife-55845-supp2.docx]

| Compound | Concentration (μM) | % binding (relative to 1,4,5-IP3) |
| --- | --- | --- |
| CAI (**9**) | 0.25  0.5  1  3  5  10 | ~0  ~0  ~0  12  3  2 |
| **10** | 0.25  0.5  1  3  5  10 | 9  6  ~0  8  ~0  ~0 |
| **11** | 0.25  0.5  1  3  5  10 | 6  ~0  ~0  ~0  10  2 |
| Mubritinib (**1**) | 0.25  0.5  1  3  5  10 | ~0  ~0  ~0  ~0  16  3 |
| 6 | 10 | 5 |

**Supplementary File 2**
